# Aeroallergens sensitization pattern in atopic patients in Qatar: A retrospective study

**DOI:** 10.5339/qmj.2023.sqac.2

**Published:** 2023-11-19

**Authors:** Sherin Thalappil, Tayseer Ibrahim, Sibi Shibu, Hassan Mobayed, Maryam Al-Nesf

**Affiliations:** ^1^Adult Allergy and Immunology Section, Department of Medicine, Hamad Medical Corporation, Doha, Qatar. Email: SThalappil@hamad.qa

## Abstract

Background: Allergic respiratory diseases (allergic rhinitis and asthma) are major health problems with high prevalence causing significant patient morbidity as well as an economic burden (1). Sensitization to inhaled allergens is a major factor in the pathogenesis of allergic respiratory diseases (2). This study aims to determine the commonest aeroallergen sensitization in patients with allergic symptoms who attended an Adult Allergy Clinic in Qatar.

Methods: This retrospective study reviewed the skin prick test results database of 20 aeroallergens performed between January 1^st^ to December 31^st^, 2022, at an Adult Allergy clinic in Qatar. Based on skin test results, the most prevalent aeroallergens were determined.

Results: A total of 554 patients (43% males, 57% females), aged between 12-87 years, 36±13.8 (mean ± SD), underwent skin prick test, of which 378 patients (68%) had positive results. There were no significant sex differences in the frequency of atopy (males: 60% versus females: 65% p= .076). Of the total 554 patients, 62% were diagnosed with Allergic rhinitis, 19% with non-allergic rhinitis, 32% with asthma, 6.7% with chronic urticaria, and 6.5% with atopic dermatitis. The frequency of sensitivity to aeroallergens was Dermatophagoides pteronyssinus 49.5%, Dermatophagoides farina 38.6%, Cat 37.3%, American Cockroach 25.9%, Russian thistle 24%, German cockroach 20%, Rough pigweed 19%, Bermuda grass 11%, and 8% to seven grass mix. 61 % were sensitized to indoor aeroallergens and 31% to different pollens (outdoor). Of the 378 patients who were sensitized, 145 patients (38%) were monosensitized, and 233 (62%) were polysensitized (≥2 allergens). There were no significant differences in the frequency of polysensitization between males and females (M:F: 1:1, p=0.938).

Conclusion: Insects (house dust mites and cockroaches) and animal protein (cat hair) were the most prevalent positive aeroallergen by skin tests. However, weed, tree pollens (Russian thistle, Rough pigweed, Mesquite tree), and grass pollens (Bermuda and seven grass mix) were also positive for a minority of patients. 62% of patients were polysensitized to aeroallergens.
